# Stretching and Compressing Capillary Bridges on Hydrophilic, Hydrophobic, and Liquid-Infused Surfaces

**DOI:** 10.1021/acs.langmuir.5c05016

**Published:** 2026-01-13

**Authors:** Sarah J. Goodband, Ke Sun, Kislon Voïtchovsky, Halim Kusumaatmaja

**Affiliations:** † Department of Physics, 3057 Durham University, Durham DH1 3LE, U.K.; ‡ Institute for Multiscale Thermofluids, School of Engineering, The University of Edinburgh, Edinburgh EH9 3FB, U.K.

## Abstract

Aqueous capillary liquid bridges are ubiquitous in nature and in technological processes. Here, we comparatively investigate capillary bridges formed between three distinct types of surfaces: (i) hydrophilic glass, (ii) hydrophobic dichlorodimethylsilane (DMS)-functionalized glass, and (iii) liquid-infused (LIS). We combine experimental measurements and computer simulations of the capillary bridges’ evolution upon changes in the gap size between the surfaces, deriving in each case the bridge geometry and the resulting capillary force. The results, also compared with predictions from the existing theory, follow expected trends on glass and DMS-functionalized surfaces: contact line pinning dominates the bridge behavior on glass with a characteristic stick–slip motion, whereas a pronounced advancing and receding hysteresis is observed on DMS surfaces. On LIS, the absence of pinning leads to minimal force variation, gravity-driven breaking of the bridge symmetry, and possible liquid exchange between LIS through bridge cloaking. These effects become particularly significant in asymmetric bridge configurations combining LIS and DMS surfaces, where the transfer of lubricant from LIS to DMS modifies the effective surface tension and alters bridge–surface interactions. Our systematic comparison of the capillary bridge behavior across solid and liquid interfaces with varying wettability provides a foundation for designing functional surface applications with controlled bridge–surface interactions.

## Graphical abstract

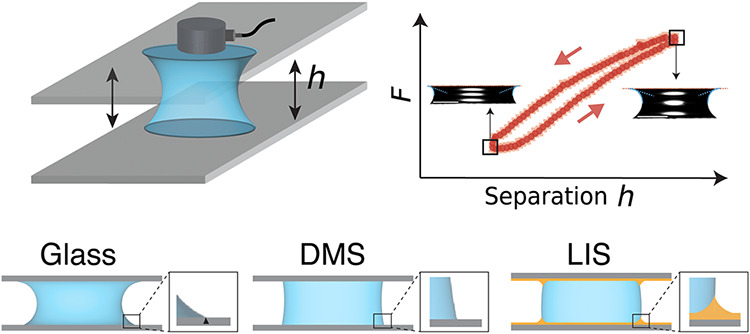

## Introduction

Capillary liquid bridges form when a liquid meniscus bridges two surfaces, generating strong adhesion forces. They are ubiquitous in nature and industry, from insect adhesion to water surfaces,^1,2^ to cohesion in soil and granular media such as sandcastles,^3,4^ semiconductor fabrication,^5^ oil recovery,^6^ cement drying,^7^ and drug delivery.^8,9^ At the microscale, these capillary forces can be dominant, also influencing macroscopic properties and temporal evolution of the system.^10–12^ The behavior of capillary bridges is governed by the liquid’s properties, bridge dimensions, the chemical and topographical characteristics of the surfaces, and environmental conditions such as temperature and humidity. These effects are reflected in extensive studies examining the impact of surface geometry,^4,13^ chemical and topographical patterning,^14,15^ wettability,^16,17^ and length scales from nanometers^18–20^ to millimeters.^21,22^ To uncover the underlying physics, studies usually quantify the bridges geometrical characteristics (e.g., contact angles (CAs), curvatures, and contact radii)^15,23,24^ and measure the capillary forces they exert^22,25,26^ when they are extended or compressed between solid surfaces.

Despite substantial progress, our understanding of capillary bridge behavior remains incomplete. Most previous studies have focused on hydrophilic solid surfaces, where contact line pinning dominates and induces hysteresis.^23,24,27^ More recently, investigations on hydrophobic surfaces with liquid features are emerging, reflecting their growing potential in applications such as self-cleaning and antifouling technologies. However, most studies hitherto employ solid surfaces, and capillary bridges between liquid-like surfaces are still largely unexplored. Liquid-infused surfaces (LIS) present a typical example of such surfaces, where porous structures are impregnated with a lubricant to achieve high liquid repellency and low friction properties.^28,29^ From a fundamental perspective, LIS represent a distinct capillary behavior regime where a three-phase fluid contact line is present. From the application side, advances in LIS and related liquid-like functional surfaces such as slippery omniphobic covalently attached liquid (SOCAL) surfaces, are critical in liquid deposition and transport in fields such as anti-icing coatings, inkjet printing, and microfluidics.^30–32^ On LIS, pinning and hysteresis are negligible,^33,34^ and the introduction of a lubricant creates more liquid–liquid and liquid–gas interfaces. This can alter capillary morphology and cause deviations from classical force models. Computational modeling by Shek et al. has shown lubricants on LIS can produce fundamentally different capillary geometries compared to solid surfaces, with increased vertical friction arising from oil ridges formed around the contact of the bridge and the surface.^35^ Furthermore, the fluid nature of LIS could induce other unexplored phenomena, such as lubricant transport between surfaces via capillary bridges.

Here, we quantitatively compare capillary bridges evolution during extension and compression on hydrophilic, hydrophobic, and LIS using a micronewton-precision experimental setup. Experiments are complemented by computational modeling that incorporates apparent contact angle to account for oil ridge formation on LIS, which is experimentally challenging to capture but crucial for influencing bridge geometry and forces. Silicon oxide glass is selected as the hydrophilic surface for its routine use and technological relevance, while DMS-functionalized hydrophobic surface and silicone-oil-infused LIS are selected for their similar wettability to isolate effects specific to the fluid nature of LIS. Comparison across these three surface types presents distinct force and geometry responses driven by phenomena such as stick–slip motion, contact angle hysteresis, and liquid ridge formation. Tests on dissimilar surface pairs of DMS and LIS further demonstrate bridge asymmetry induced by gravity, as well as lubricant transfer from LIS to the opposing surface. Overall, we present a systematic experimental and computational study that establishes a benchmark for understanding and predicting capillary bridge evolution on solid and liquid functional surfaces, offering mechanistic insights to the rational design of surfaces with liquid features.

## Methods

### Surface Preparation

#### Hydrophilic Surface – Glass

Silicon oxide glass coverslips (25 mm × 25 mm, thickness 0.13–0.16 mm, VWR, UK) are used directly from a freshly opened box without additional cleaning procedures to ensure chemical stability during measurements. We characterized the surface roughness using atomic force microscopy (AFM), obtaining a mean surface roughness *S*_a_ = 0.449 ± 0.027 nm, a root-mean-square roughness of *S*_q_ = 1.031 ± 0.207 nm, and a surface area *A* to projected area *A*_0_ ratio of *A*/*A*_0_ = 1.00039 ± 0.00008 (see details in Supporting Information SI 1).

#### Hydrophobic Surface – DMS

Hydrophobic surfaces are prepared by chemical vapor deposition (CVD) of dichlorodimethylsilane (DMS) on glass coverslips (reference hydrophilic surfaces).^36^ Glass slides are sequentially cleaned by acetone (99%, Sigma-Aldrich, UK) and isopropanol (99.8%, Fisher Scientific, UK), followed by 30 min of sonication. They are then dried under nitrogen and plasma-cleaned for 10 min (>30 W, VacuLAB-X, UK) and are dehydrated in an oven at 100 °C for 1 h. For CVD, 1 mL of DMS is placed in an open dish in a desiccator along with the slides directly transferred from the oven and kept under vacuum overnight. Finally, the slides are rinsed with acetone and ultrapure water (18.2 MΩ, Merck-Millipore, UK), then dried at 40 °C overnight. AFM measurements of the DMS-functionalized surface yield *S*_a_ = 2.297 ± 0.221 nm, *S*_q_ = 3.427 ± 0.380 nm, and *A*/*A*_0_ = 1.00037 ± 0.00007 (see details in Supporting Information SI 1).

#### LIS

LIS are prepared following established protocols.^37,38^ In short, glass slides are first cleaned by soaking in an aqueous solution of Decon 90 (Decon Laboratories Ltd., UK) before rinsing and sonicating in ultrapure water to remove residual detergent, followed by air-drying. Prior to coating, slides are rinsed with acetone and isopropanol and dried under nitrogen and then air-dried. Five layers of nanoparticles are then sequentially applied to the surface using a liquid spray (GLACO, SOFT 99 Corp.) with 1 h interval between layers. A drop of 50 μL silicone oil (20 cSt @ 25 °C, density ρ = 0.95 g mL^–1^, surface tension γ = 20.6 mN m^–1^ in air, Sigma-Aldrich, UK) is then placed on the surface and spin-coated (2000 rpm, 5 min). The slides are used immediately or stored without oil coating, in closed Petri dishes for a maximum of 2 weeks. LIS fabricated with this protocol retains a stable oil layer thickness (>3 μm) with no exposed nanoparticles. This allows the LIS to maintain their chemical and wetting properties over the time scale of the experiments,^38^ as also evidenced by the negligible hysteresis reported in the Results section.

### Capillary Bridge Measurement and Error Control

A detailed description of the experimental protocol, apparatus, and data processing procedure is provided in Goodband et al.^39^ In brief, the capillary bridge is formed between two parallel substrates, with the top plate attached to a force sensor and the bottom plate mounted on a motorized stage used to compress and stretch the bridge. The system is imaged using a dual camera arrangement: one camera focuses on the bridge edge to provide high resolution profiles for extracting geometrical parameters, while a second synchronized camera views the bridge from another side to support the analysis (see an illustration in Supporting Information SI 2). We maintain identical capillary bridge volume and composition across all experiments to ensure direct comparison.

Experiments are conducted under ambient laboratory conditions (20–23 °C, relative humidity 60–70%). To start with, two solid surfaces prepared using the above protocols are mounted onto a custom-built plate using an adhesive (Reprorubber, Bowers Group, UK) and are allowed to cure for 2 h before the plate is mounted to the force sensor. The force sensor is then equilibrated for 1 h before measurements. To ensure protocol consistency and data reproducibility, a 10 μL droplet of 80 wt % glycerol in ultrapure water (used to limit evaporation) is placed always onto the bottom surface and is gently brought into contact with the top surface (see details in Supporting Information SI 3), followed by equilibration for 2 min. The droplet has a measured density ρ = 1.20 g mL^–1^, a viscosity η = 45.35 mPa s (from Moreno-Labela et al.^40^), and a measured surface tension γ of 67.2 mN m^–1^ in air and 27.6 mN m^–1^ in 20 cSt silicone oil. During measurements, the bridge is first extended and then compressed at a constant rate of 0.008 mm s^–1^, with a maximum separation difference of 0.5 mm between the most compressed and most stretched states.

Accurate tracking of the capillary bridge contact line is important for understanding pinning effects but is experimentally challenging to achieve simultaneously at both the top and bottom interfaces due to limitations in optical focus (see Supporting Information SI 4). However, capturing information at both extremities is necessary for quantifying the gravitational effects, probing asymmetric bridges, and evaluating the experimental approach against theoretical predictions. In practice, we acquire top and bottom measurements separately by readjusting camera to capture top and bottom in subsequent extension-compression cycles, with control experiments confirming that the measured values do not vary significantly (see Supporting Information SI 5).

Even following the same protocol, variations in the measured geometrical parameters can be observed between data sets. For instance, changes in ambient humidity and temperature can result in CA variations of ∼2° for a LIS sample,^38^ and DMS surfaces are observed to have up to 3 °CA differences following the same protocol. In all cases, the measurements are conducted over a few hours to minimize environmental impact and ensure highly consistent data sets.

### Capillary Force Calculation

To quantify the capillary force exerted by a capillary bridge, it is necessary to measure its geometry throughout the experiment. Figure 1 shows the key geometrical parameters of a capillary bridge formed between two parallel substrates. Forces are measured exclusively on the top substrate, but they can theoretically be calculated for both.

**Figure 1:**
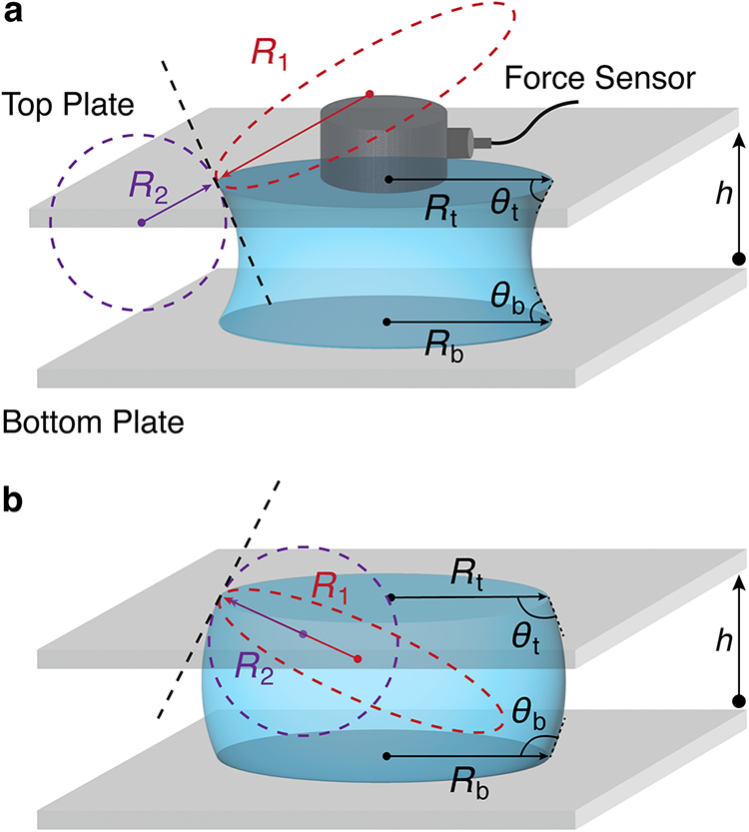
Schematics of concave (a) and convex (b) capillary bridges between two parallel plates separated by a distance *h*. θ_t_ and θ_b_ denote the contact angles at the top and bottom of the bridge, *R*_t_ and *R*_b_ are the corresponding top and bottom contact radii. The mean curvature of the bridge is determined from the azimuthal (*R*_1_) and meridional (*R*_2_) radii of curvature. *R*_1_ and *R*_2_ are obtained orthogonally at either the top (illustrated here) or the bottom of the bridge, depending on which plate the force is being calculated for (see details in the force derivation and Supporting Information SI 6).

When gravity is negligible, e.g., for a bridge much smaller than the capillary length, the equilibrium capillary force *F* between two identical parallel plates can be expressed by the direct action of surface tension and Laplace pressure:^11,35^

1 $$F=-2\pi \gamma R\;\sin\left(\theta \right)+\pi R^{2}\Delta P$$

where γ is the liquid surface tension, *R* is contact radius, θ is the contact angle, and Δ*P* is the Laplace pressure between the bridge and surrounding fluid. When gravity is negligible, the top and bottom contact angles and contact radii are equal. When gravity cannot be neglected, the capillary bridge becomes asymmetric, and forces exerted by the top and bottom differ. Following the Young–Laplace equation, the capillary force on the top plate can be calculated from the geometrical parameters at the top:

2 $$F_{t}^{\mathrm{calc}}=-2\pi \gamma R_{t}\;\sin\left(\theta_{t}\right)+\pi {R_{t}}^{2}\gamma \left(\frac{1}{R_{1}}+\frac{1}{R_{2}}\right)$$

where *R*_t_ and θ_t_ are the top contact radius and contact angle. The radii of curvature *R*_1_ and *R*_2_ correspond to the azimuthal and meridional radius of curvature, respectively. For a given plate (top or bottom), the two corresponding radii are measured orthogonally at that plate: *R*_2_ is obtained by fitting the local bridge edge to a second order polynomial, and *R*_1_ is determined from the geometry of the three-phase contact line (see details in Supporting Information SI 6).^39^ For a concave capillary bridge, *R*_1_ is positive and *R*_2_ is negative; for a convex capillary bridge, both *R*_1_ and *R*_2_ are positive. Hereafter, this equation will be called the “top calculated force” *F*_t_^calc^. Similarly, the bottom capillary force can be expressed as

3 $$F_{b}^{\mathrm{calc}}=-2\pi \gamma R_{b}\;\sin\left(\theta_{b}\right)+\pi {R_{b}}^{2}\gamma \left(\frac{1}{R_{1}}+\frac{1}{R_{2}}\right)$$

where *R*_b_ and θ_b_ are the bottom contact radius and contact angle. Since this expression relies on the bottom measured parameters, it will hereafter be called the “bottom calculated force” *F*_b_^calc^. In eqs 2–3, θ_t_ and θ_b_ denote the Young’s contact angles on solid substrates, or the apparent contact angles in the presence of oil ridges, as defined in Computational model section.

In the experimental setup, a force sensor is implemented on the top plate to acquire a “top measured force”, *F*_t_^meas^. While the bottom force cannot be measured directly, it can be easily inferred from the top measured force by accounting for gravity:

4 $$F_{b}^{\inf}=F_{t}^{\mathrm{meas}}+\rho \mathrm{gV}$$

where ρ is the density of the droplet, *V* is the capillary bridge volume, and *g* is the gravitational acceleration. Since this approach relies on the top measured force to infer the bottom force, it will be referred to as the “bottom inferred force” *F*_b_^inf^. Equations 3 and 4 thus provide two complementary methods to determine the bottom force, the advantages of which are discussed in detail in the Results section.

### Computational Model

We employ quasistatic simulation using the Surface Evolver^41^ software. In brief, the model incorporates the fluid and solid interfaces, with vertices relax in a gradient descent manner to reach the system’s minimum energy configuration. As the capillary bridges considered here are comparable in size to the bridge liquid’s capillary length, gravity is incorporated into the model by matching the Bond number to the experimental value (see details in Supporting Information SI 7). For simple solid surfaces (Glass and DMS), we initialize the droplets in between two plates, using experimentally measured droplet–gas interfacial tension γ_dg_ and the bottom Young’s contact angle θ_b_ (Figure 2a). The rest of interfacial tensions are related via Young’s equation, γ_ds_ = γ_sg_ – γ_dg_ cosθ_b_, where γ_ds_, γ_sg_ are the droplet–solid and solid–gas interfacial energies, respectively. For cases involving LIS or lubricant transfer from LIS to DMS, the Young’s contact angle on a solid surface is no longer applicable. Instead, we define an apparent droplet contact angle, such as θ_b,app_ in Figure 2b, obtained by measuring the angle between the droplet–air interface profile and the horizontal plane. Here, we take the bottom plate as an example; a similar approach can be applied to the top surface when an oil ridge is present. In such situations, oil ridges form around the capillary bridge at its contact with the plate, giving rise to a Neumann triangle at the oil–droplet–air three-phase interface (Figure 2b, inset). Although these features are too small to resolve experimentally, simulations that incorporate the relevant interfacial tensions can infer the three phase contact geometry. Specifically, the interfacial tensions γ_od_ (oil–droplet), γ_dg_ (droplet–gas), γ_og_ (oil–gas) are obtained from pendant drop measurements. The oil–gas contact angles θ_og_ is either assumed from the intrinsic oil wettability on the substrate or independently determined from lubricant cloaking measurements (Supporting Information SI 8). These quantities are related through the following expression:^35^

5 $$\cos\theta_{b,\mathrm{app}}=-\cos\theta_{\mathrm{od}}\frac{\gamma_{\mathrm{od}}}{\gamma_{\mathrm{dg}}}+\cos\theta_{\mathrm{og}}\frac{\gamma_{\mathrm{og}}}{\gamma_{\mathrm{dg}}}$$

**Figure 2:**
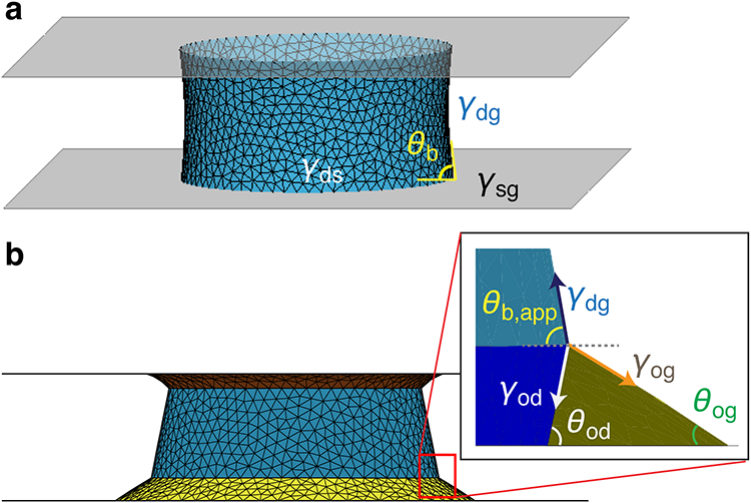
Simulation snapshots of capillary bridges between two solid parallel plates (a) and between plates with oil rings (b). γ_ds_, γ_dg_, γ_sg_, γ_od_, and γ_og_ are the interfacial tensions of the droplet–solid, droplet–gas, solid–gas, oil–droplet, and oil–gas interfaces, respectively. θ_b_ in (a) is the Young’s contact angle of the capillary bridge on the bottom solid plate, and θ_b,app_ in (b) is the apparent contact angle of the bridge on the bottom plate when surrounded by an oil ridge. The inset in (b) illustrates the Neumann triangle at the droplet–air–oil contact, with γ_dg_, γ_og_, and γ_od_ representing the interfacial tensions that satisfy the force balance. At the bottom substrate, θ_og_ denotes the oil–air contact angle at the plate, and θ_od_ denotes the oil–droplet contact angle at the plate. The schematics in (a) and (b) explicitly illustrate the bottom plate; the same conventions apply to the top plate.

Equation 5 allows θ_od_ to be derived once θ_b,app_, θ_og_, and the interfacial tensions are known. The resulting values of θ_od_, together with the measured interfacial tensions and a prescribed oil ridge volume, are then used as inputs for the simulation model. For the systems with capillary bridge between parallel LIS surfaces, θ_od_ remains essentially constant with only minor variation (∼2–3°) during compression and extension. In DMS-LIS systems, the LIS side again shows little change, whereas the DMS side varies strongly, by ∼30° between the most compressed and extended configurations due to lubricant transfer and ridge pinning. The actual size of the oil ridges depends on factors such as lubricant pressure and oil thickness, which could not be fully captured in the current experimental setup. To satisfy simulation resolution and system symmetry, the oil ridges input in the model may therefore be larger than those in the experiments. Nevertheless, this approximation is based on experimental measurements and yields good agreement between simulated and experimentally measured capillary bridges, indicating that both the oil ridges and the three-phase contact are accurately captured by the model (see Results section).

The simulations also allow calculation of the exerted capillary force. For convenience, here we evaluate it at the bottom contact line between the droplet and the oil ridge. Adapted from eq 1, the force is given by

6 $$F_{\mathrm{sim}}=-2\pi \gamma_{\mathrm{dg}}R_{\mathrm{cl}}\;\sin\left(\theta_{b,\mathrm{app}}\right)+\pi {R_{\mathrm{cl}}}^{2}\Delta P$$

where *R*_cl_ is the is the radius of the circular three-phase contact line (bridge–oil–air), and Δ*P* is the pressure difference across the droplet body, obtained directly from the simulation output. This formulation links the simulated interfacial geometry to the measurable capillary force, and the resulting predictions are in good agreement with experimental measurements (see Results section). Having clarified how the macroscopic contact angle is measured at for the bridge at both solid surfaces and surfaces with oil ridges, we hereafter simply refer to the angle as θ_t_ and θ_b_, regardless of whether it corresponds to the Young’s contact angle on a solid substrate or the apparent contact angle in the presence of an oil ridge.

## Results and Discussions

### Capillary Bridge between Identical Parallel Surfaces

To build a basic understanding of the capillary behavior on distinct surfaces, we begin by comparing capillary bridges between identical top and bottom surfaces, ranging from the hydrophilic Glass surface, to the hydrophobic DMS-functionalized surface, and the LIS. For clarity, hydrophilic Glass surface and DMS-functionalized surface will hereafter be referred to as “Glass” and “DMS” respectively. Upon extension and compression of the capillary bridges, we simultaneously measure and calculate geometrical parameters and exerted forces. Figure 3a–c present the most compressed and most stretched geometries of the capillary bridge, showing good agreement between experiments and simulations for all cases. The bridge geometry differs across surface types, exhibiting convex or concave shapes during extension and compression. To quantify these geometrical variations, we track the evolution of the contact angles, contact radii, and the meridional curvature as a function of plate separation.

**Figure 3:**
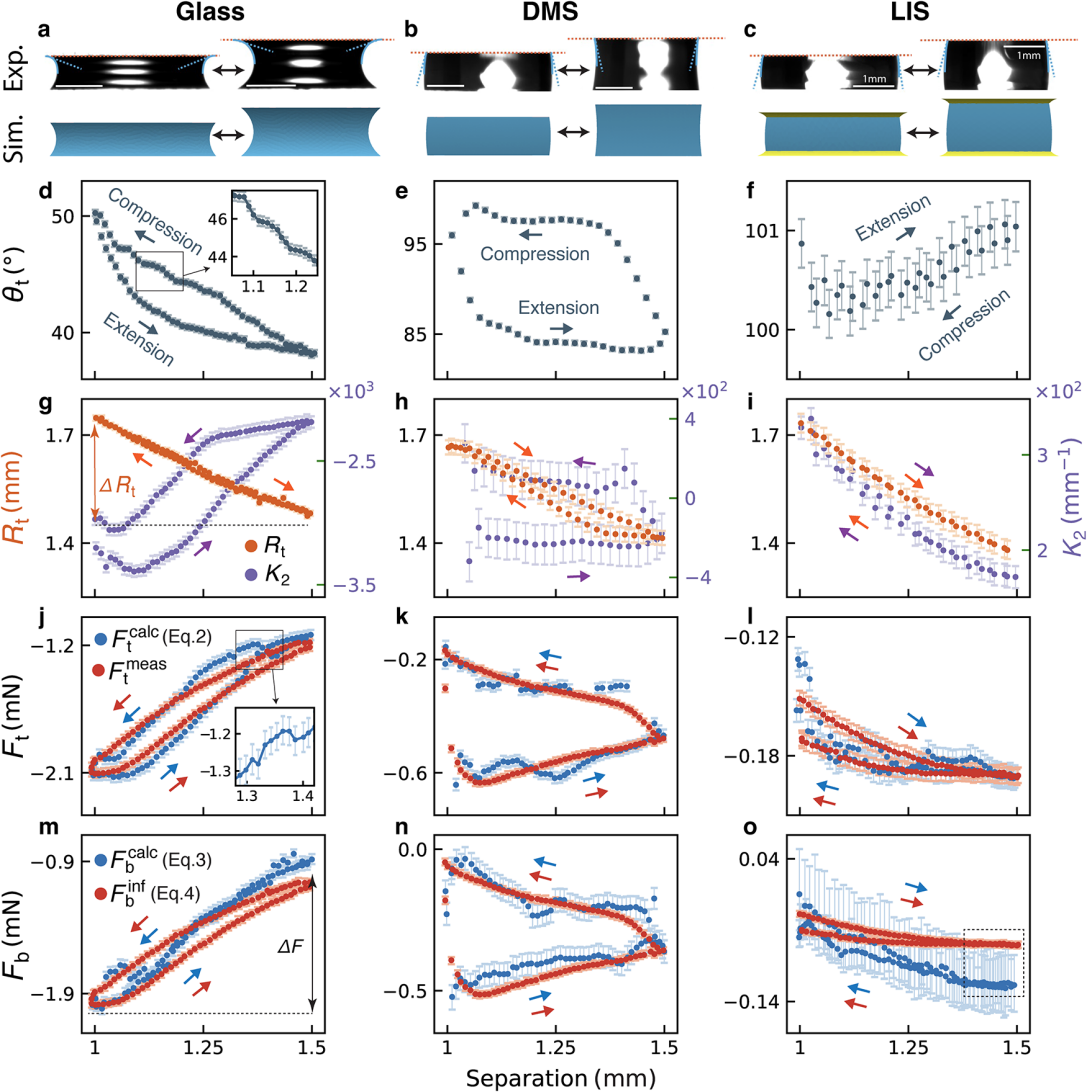
Geometry and force comparison of capillary bridges between identical top and bottom surfaces: hydrophilic glass surface, hydrophobic DMS-functionalized surface, and Liquid-infused surfaces (LIS). (a–c) Capillary bridge geometries in the most compressed and most stretched state. Evolution of the geometrical features is shown for top contact angles θ_t_ (d–f), top contact radius *R*_t_, and meridional curvature *K*_2_ (g–i, *K*_2_ = 1/*R*_2_ and *R*_2_ is the meridional radius of curvature in Figure 1). The change in contact radius within an extension-compression cycle, Δ*R*_t_, is marked in (g) as an example. Panels (j–l) compare the measured (*F*_t_^meas^, red) and calculated force (*F*_t_^calc^, blue) for the top surface, while panels (m–o) compare the inferred (*F*_b_^inf^, red) and calculated force (*F*_b_^calc^, blue) for the bottom surface. Arrows indicate the extension-compression direction in each panel, with Δ*F* denoting the force variation in the process, as shown in (m). Insets in (d) and (j) highlight the stepwise stick–slip features in the measured contact angles and forces. The dashed square in (o) marks the deviation between the inferred and the calculated bottom forces, particularly at larger plate separations. Error bars represent standard errors.

We first discuss the top contact angle θ_t_ and top contact radius *R*_t_. On Glass, θ_t_ decreases as the plate separation increases, spanning a hydrophilic range of 38–50° in conjunction with contact line motion (Figure 3d,g). Hysteresis is observed between extension and compression cycles. Notably, a stepwise increase in θ_t_ occurs during capillary bridge compression (Figure 3d, inset), without dominant plateaus as the contact line advances or recedes. However, this stepwise feature is not observed in the contact radius *R*_t_ (Figure 3g). This observation indicates a complicated stick–slip behavior involving alternating pinning and rapid movements of the contact line, likely due to small and asymmetric local pinning points as the evolutions of contact angle and radius are not straightforwardly correlated. The effect of pinning is particularly evident when comparing the change in contact radius Δ*R*_t_. Glass exhibits the smallest contact radius variation (Δ*R*_t_ = 0.27 mm, Figure 3g), whereas LIS, which exhibits no pinning, shows the largest Δ*R*_t_ = 0.4 mm (Figure 3i). For DMS, the CA exhibits typical hysteresis expected for hydrophobic surfaces,^23,27,42,43^ with a hydrophobic (>90°) advancing CA at ∼98° and a hydrophilic (<90°) receding CA at ∼84°, corresponding to the plateaus in CA observed during compression and extension, respectively (Figure 3e). In contrast, LIS benefits from its low friction liquid characteristics, yielding a highly stable θ_t_ with negligible hysteresis within experimental error (Figure 3f). This observation aligns with other studies^44,45^ using silicone oil as the lubricant, which also report low contact angle hysteresis on LIS. During extension, the slight increase in θ_t_ at larger separation distance (Figure 3f) can be attributed to interactions between the capillary bridge and the LIS lubricant ridge. This is addressed further in Figure 5 and associated text.

The meridional curvature *K*_2_ (*K*_2_ = 1/*R*_2_, where *R*_2_ is the meridional radius of curvature in Figure 1) is closely related to the CA and the overall capillary bridge geometry. Among the three cases, the capillary bridge on Glass experiences the highest curvature at ∼ −3000 mm^–1^ on average (Figure 3g), due to its low CA and significant contact line pinning. The curvature remains negative, thus the capillary bridge on Glass retains a concave shape during extension and compression. On DMS, θ_t_ crosses between the hydrophilic and hydrophobic regimes at 90°, resulting in both positive and negative curvatures. The capillary bridge is concave when most stretched and convex when most compressed (Figure 3b,h). As a hydrophobic surface with similar CA, the meridional curvature of the capillary bridge on LIS is of similar magnitude to that on DMS but remains positive, reflecting the relatively constant θ_t_ and negligible hysteresis (Figure 3i). Overall, curvature hysteresis is highest on Glass, intermediate on DMS, and negligible on LIS, consistent with the observed contact angle hysteresis.

Aside from the geometrical parameters presented in Figure 3d–i, it is useful to consider the symmetry of the contact line during an extension-compression cycle, as capillary bridges do not necessarily move symmetrically when pinning occurs. This can be quantified by tracking the displacement of the contact points at the top and bottom of the capillary bridge with the solid plates after full extension-compression cycles, thereby allowing quantification of asymmetry on both sides. Only small displacements are observed for Glass and LIS due to strong pinning in the former and frictionless motion in the latter. In contrast, DMS exhibits a much larger asymmetry, resulting from a combination of pinning and contact line displacement (see details in Supporting Information SI 9). These observations are consistent with the expected behavior for each system, highlighting the various phenomena at play in capillary bridge behavior.

From the geometrical parameter evolution during compression and extension, we can now calculate the associated capillary force exerted by the substrates using eqs 2–4. The forces calculated from geometrical measurements are denoted as *F*_t_^calc^ (eq 2) and *F*_b_^calc^ (eq 3) for top and bottom surfaces, respectively. The force directly measured by the force sensor on the top surface is denoted as *F*_t_^meas^, while the bottom force is inferred by adding a gravity term, and denoted as *F*_b_^inf^ (eq 4). Figure 3j–o compare measured or inferred forces with the calculated values for each system. Generally, the top measured force *F*_t_^meas^ agrees well with the calculated force *F*_t_^calc^ within experimental error, including on LIS (Figure 3j–l). This suggests that capillary theory developed for solid surfaces can be readily adapted to predict the capillary forces on LIS, at least in the limit of a small lubricant ridge considered in this work.

The magnitude of the capillary force, however, varies significantly across these different systems. On Glass, the stronger interactions between the capillary bridge and the surface yield an absolute force value of *F* ∼ 2 mN with a variation of Δ*F* ∼ 1 mN over an extension-compression cycle (Figure 3j,m). In contrast, the force magnitudes on the DMS and LIS are considerably smaller, on the order of 0.1 mN. Notably, the force variation over an extension-compression cycle is five times larger for DMS (Δ*F* ∼ 0.4 mN, Figure 3k,n) than for LIS (Δ*F* ∼ 0.06 mN, Figure 3l,o), thanks to the frictionless nature of LIS. On Glass and DMS, occasional small deviations between the calculated and measured forces are observed, arising from pinning events that cannot be easily captured experimentally, as pinned points may lie outside of view.

The bottom inferred force *F*_b_^inf^ and calculated force *F*_b_^calc^ still agree within error (Figure 3m–o). However, it is noticeable that agreement is poorer for the bottom than the top of the bridge. This is to some extent expected since the comparison is less direct than at the top surface. On Glass and DMS, *F*_b_^inf^ and *F*_b_^calc^ show good agreement, with an overall behavior of simply shifted version from the top surface data. On LIS, however, a noticeable difference is observed between the inferred and calculated force (see dashed square in Figure 3o), and several factors contribute to this complex comparison. First, the exerted forces on LIS are considerably smaller than those on Glass or DMS, making the relative errors inevitably larger (Figure 3m–o). Second, there are uncertainties in the surface tension used to calculate *F*_b_^calc^ (eq 3), as the capillary bridge is likely to be cloaked by the LIS lubricant. Cloaking is a well-known phenomenon in LIS.^46–48^ Here, we estimate the spreading coefficient of the lubricant over the capillary bridge to be *S* ∼ 20 mN/m, suggesting full cloaking. Determining an effective surface tension for the cloaked capillary bridge is not straightforward as the surface tension of a thin film is known to vary with its thickness.^49^ In this setup, we are unable to measure the thickness of the cloaking film which may not be uniform and may evolve over the course of an experiment, and lubricant transport between the two surfaces through the capillary bridge is possible. To reflect this uncertainty, we adopt an effective droplet–gas surface tension of γ_dg_ = 50 ± 2 mN m^–1^, obtained by averaging our pendant drop measurement (analyzed by the Opendrop software^50,51^ ) with a value inferred from literature data for a similar system^52^ (see Supporting Information SI 10 for details). While this reduction from the uncloaked droplet’s surface tension (∼67 mN m^–1^) to the effective cloaked value (∼50 mN m^–1^) is relatively small, it is sufficient to significantly affect the data considering the small forces at play (∼20–25% of the force value). Finally, the presence of a lubricant ridge around the capillary bridge on LIS further complicates the measurement of the meridional radius of curvature *R*_2_, which is required for calculating *F*_b_^calc^ (see Supporting Information SI 11 for more details).

The above observations and the analysis of the bridges’ geometrical features and exerted forces raise three immediate questions: (i) For solid surfaces with roughness or chemical heterogeneities, how can the stick–slip motion of the contact line be described, and what is its impact on geometry and force? (ii) Why does LIS show poorer bottom force comparison between inferred and calculated force compared to Glass and DMS surfaces? (iii) Both DMS and LIS are hydrophobic surfaces but have distinct capillary behaviors. If combined in a single capillary bridge, which behavior would dominate?

### Simulation of Stick–Slip Motion on Heterogeneous Surfaces

Experimentally, investigating the stick–slip motion in capillary bridges is challenging due to the asymmetric and highly localized nature of contact line pinning. To gain better insights into the underlying mechanisms, we performed numerical simulations examining the bridge geometry evolution and capillary force dynamics during compression and extension cycles over chemically heterogeneous surfaces. Practically, we use a binary-patterned substrate featuring alternating regions of high and low contact angles (50 and 40°, respectively) to systematically model surface chemical heterogeneity (Figure 4a). For simplicity, we ignore gravity in the simulations since it is not critical for the contact line pinning-depinning behavior. During compression (Figure 4b), the three-phase contact line advances until it encounters a boundary transitioning from low-CA to high-CA regions, where strong pinning occurs. At this stage, the contact radius *R* remains fixed while the bridge height continues to decrease under the applied compression, resulting in an increase in the measured CA. Once the local contact angle reaches the prescribed high-CA value, the pinning constraint is released, allowing the contact line to advance across the high-CA region. When reaching the subsequent low-CA region, the contact line exhibits rapid forward motion due to the energetically favorable wetting conditions. This alternating sequence of pinning and release events repeats throughout the compression process. During stretching, the process is reversed. The receding contact line becomes preferentially pinned at boundaries transitioning toward low-CA regions, where higher wettability disadvantages receding. This pinning behavior during both compression and extension cycles generates distinctive stepwise variations and hysteresis both in the *R* and CA evolutions (Figure 4c,d), providing clear experimental signatures of the stick–slip phenomenon.

**Figure 4:**
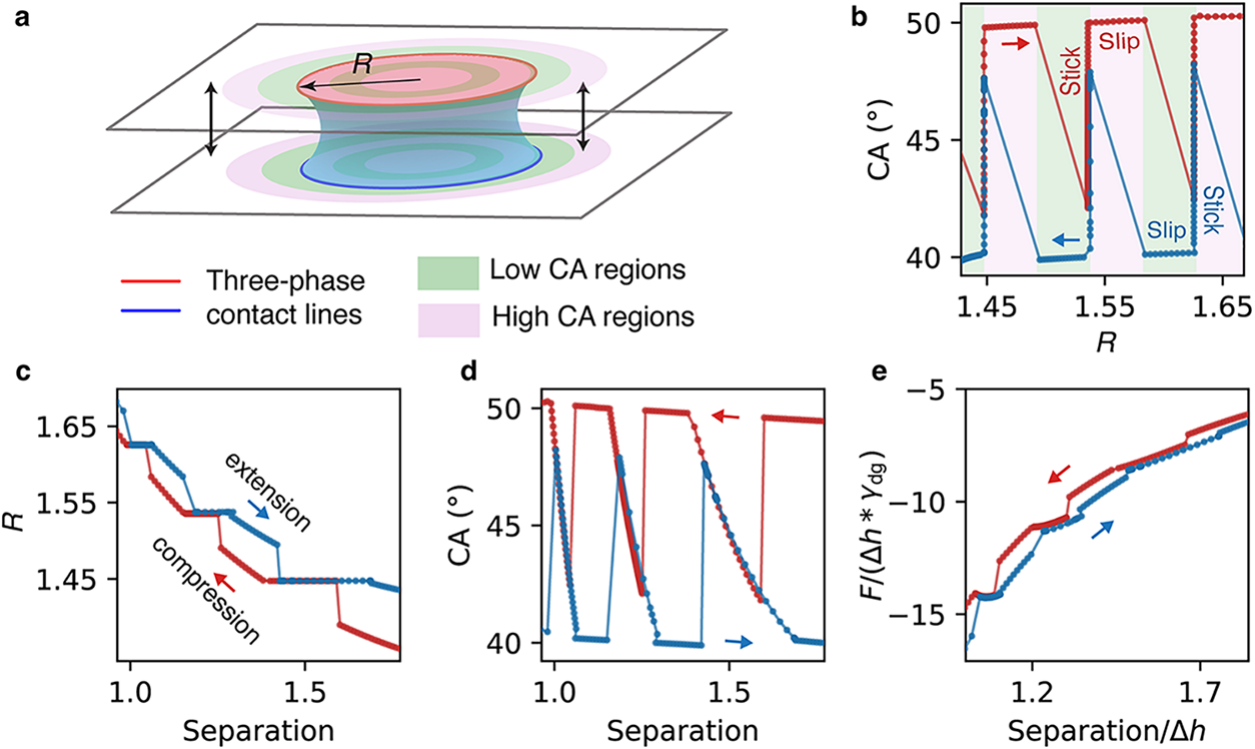
Simulated capillary bridge behavior on a binary-patterned surface featuring alternating high-CA (purple, 50°) and low-CA (green, 40°) rings. The three-phase contact line between the bridge, air, and solid are highlighted in red and blue in (a). The motion of the contact line across the heterogeneous surface exhibits stick–slip behavior, with the corresponding geometric parameters (contact radius R, and contact angle CA measured near the contact line) and capillary forces shown in (b–e). Gravitational effects are neglected to ensure symmetric contact with both patterned plates, resulting in equal top and bottom contact radii. Δ*h* denotes the maximum plate separation during the compression–extension cycle, and γ_*dg*_ is the droplet–gas interfacial tension. Because the correlation between bridge separation and base radius is nonlinear, (b) additionally shows the CA plotted against *R* to directly reflect the imposed binary pattern, whereas (c–e) present all results as a function of separation for consistent comparison with Figure 3.

The capillary force in the above simulation was calculated by eq 1 with the pressure obtained from the simulation model and normalized by the product of the plate separation change Δ*h* and the droplet–gas interfacial tension γ_dg_. The resulting force (Figure 4e) exhibits a stepwise behavior similar to that visible for Glass in Figure 3j. Similarly, it is possible to simulate roughness-induced contact line pinning and depinning, also inducing stepwise features in force and geometry measurements (see details in Supporting Information SI 12). These results confirm that ability of simulations to capture the fundamental aspects of the stick–slip hysteresis on binary-patterned and rough surfaces, and offer a basis for studying more complex substrate designs and interfacial interactions.

### Top and Bottom Symmetry of the Capillary Bridges

As discussed in Capillary bridge between identical parallel surfaces section, the discrepancy between the bottom inferred force *F*_b_^inf^ and calculated force *F*_b_^calc^ of LIS can be attributed to uncertainty in the parameters used to obtain *F*_b_^calc^ (eq 3). For capillary bridge on LIS, the capillary shape can be distorted due to differences in the top and bottom lubricant menisci that can modify local interfacial stresses. Also, the bridge–air interface may be nonideal due to the presence of a cloaking lubricant film. Consequently, *F*_b_^calc^ is a local approximation that may not capture the true force in asymmetric or complex interfaces.

To examine such symmetry effects, we analyze the evolution of the CAs at the top and bottom surfaces for Glass, DMS, and LIS systems (Figure 5). On Glass, the top and bottom CA show similar monotonic decrease upon extension, consistent with contact line pinning and stick–slip motion (Figure 5a). On DMS, both surfaces exhibit the characteristic hysteresis loop discussed in Capillary bridge between identical parallel surfaces section (Figure 5b). For LIS, the CAs on both surfaces increase by 2–3° during extension with angles overlapping during outward and return motions, indicating the absence of hysteresis. As the capillary bridge is stretched on LIS, the droplet Laplace pressure decreases, explaining the increase in the CA.

**Figure 5:**
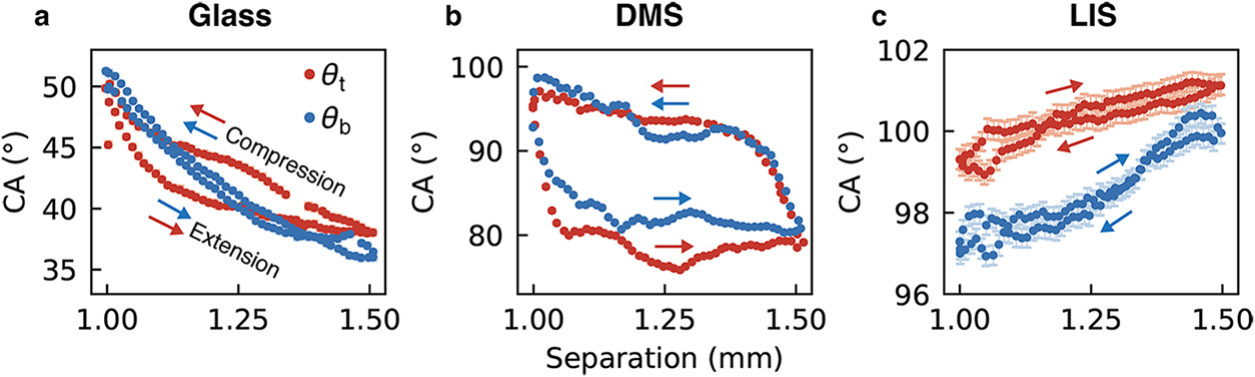
Variations in the capillary bridges’ top contact angle θ_*t*_ (red) and bottom contact angle θ_*b*_ (blue) at their contact with Glass (a), DMS (b), and LIS (c). Error bars represent two standard errors and may not be visible on Glass or DMS. The data shown comes from a different set than that presented in Figure 3 but is obtained following an identical protocol.

Notably, a small but consistent offset (∼2°) exists between the top and bottom CAs, θ_t_ and θ_*b*_ (Figure 5c). Unlike pinning-induced asymmetry, this offset originates from the pressure ratio between the bridge and the lubricant.^53,54^ For the typical capillary bridge considered in this work, the hydrostatic pressure difference between the top and bottom of the bridge is sufficient to account for the observed CA asymmetry. The maximum capillary force on LIS is around 5 to 10 times lower than for DMS and Glass (Figure 3j–o), making the geometry more sensitive to gravitational effects. The maximum bottom force for LIS is ∼0.15 mN (Figure 3o), while the gravity term ρ*gV* (eq 4) for such capillary bridge is ∼ 0.12 mN, indicating gravity and surface tension effects become comparable. In contrast, capillary bridges on Glass and DMS formed by the same droplet reach maximum forces of 0.7–2.2 mN (Figure 3m,n), where surface tension remains dominant throughout most of the extension-compression cycle.

### Asymmetric Hydrophobic Capillary Bridge with DMS and LIS by Design

So far, our results have focused on capillary bridges formed between identical top and bottom surfaces. Moving from Glass to DMS to LIS, the surfaces become progressively more hydrophobic, with increasing CAs. Glass and DMS represent widely used bare or functionalized solid surfaces, where the capillary bridge behavior is dominated by contact line pinning. LIS, in contrast, exhibits distinct behavior arising from the lubricant’s fluid nature, characterized by low friction, dynamic menisci, and low exerted forces. The capillary bridge on LIS is not perfectly symmetric at the top and bottom, although the effects of asymmetry are generally subtle. To explore this further, we design experiments with deliberately asymmetric systems, using different surfaces at the top and bottom. To prevent one surface from dominating, it is helpful to retain some similarity by selecting surfaces of comparable hydrophobicity. Here, we do this by using DMS and LIS surfaces, with each surface alternately positioned on the top or bottom. This asymmetric system is interesting because it represents two hydrophobic surfaces: one a solid surface that exhibits typical hysteresis and pinning, and the other a liquid-infused surface which is smooth, frictionless, and dynamically adaptive.

Figure 6 shows the results of designed asymmetric capillary bridge. The CA measured on the LIS remains almost constant at 98–100°, regardless of whether LIS is placed at the top or bottom (Figure 6c,i, red traces). The small difference in CA between the two configurations arises from the gravitational deformation of the bridge, consistent with the behavior observed in the symmetric system (Figure 5c). However, the evolution of the CA on the DMS surfaces is markedly different from that observed in the symmetric DMS system. In the symmetric DMS case, pinning induces a CA hysteresis loop with two plateaus, an advancing angle of ∼ 98° and a receding angle of ∼84° (Figures 3e, 5b). In the asymmetric case here, this hysteresis loop becomes elongated. The CA changes monotonically with larger absolute variations (Figure 6c,i, blue traces), and without stabilizing at the typical advancing or receding plateaus. This behavior originates from two concurrent effects. First, the surface tension of the capillary bridge is altered because the bridge is cloaked by the lubricant. Second, lubricant is transferred from the LIS to the DMS surface. This lubricant accumulation forms a ridge at the bridge–DMS contact line, which broadens the accessible range of CAs. To probe this effect independently, we perform droplet experiments on DMS under cycles of volume variation as lubricant diffuses slowly toward it (see SI 8 and Video S1). Initially, we can identify advancing and receding angles in agreement with Figures 3e and 5b for the capillary bridge setup, as the lubricant has no or very limited contact with the droplet. As the lubricant progressively wets the droplets, a ridge forms at the contact line on DMS. This ridge pins the droplet and produces larger droplet CA variation than observed before lubricant contact. To further verify that lubricant can migrate from the LIS to the DMS during capillary bridge deformation, we perform an experiment using a dyed silicone-oil-infused LIS (bottom) and a DMS surface (top). During bridge compression, we can observe the dyed lubricant migrate along the bridge to the DMS surface (see details in Supporting Information SI 13), providing direct qualitative evidence of lubricant transfer.

**Figure 6:**
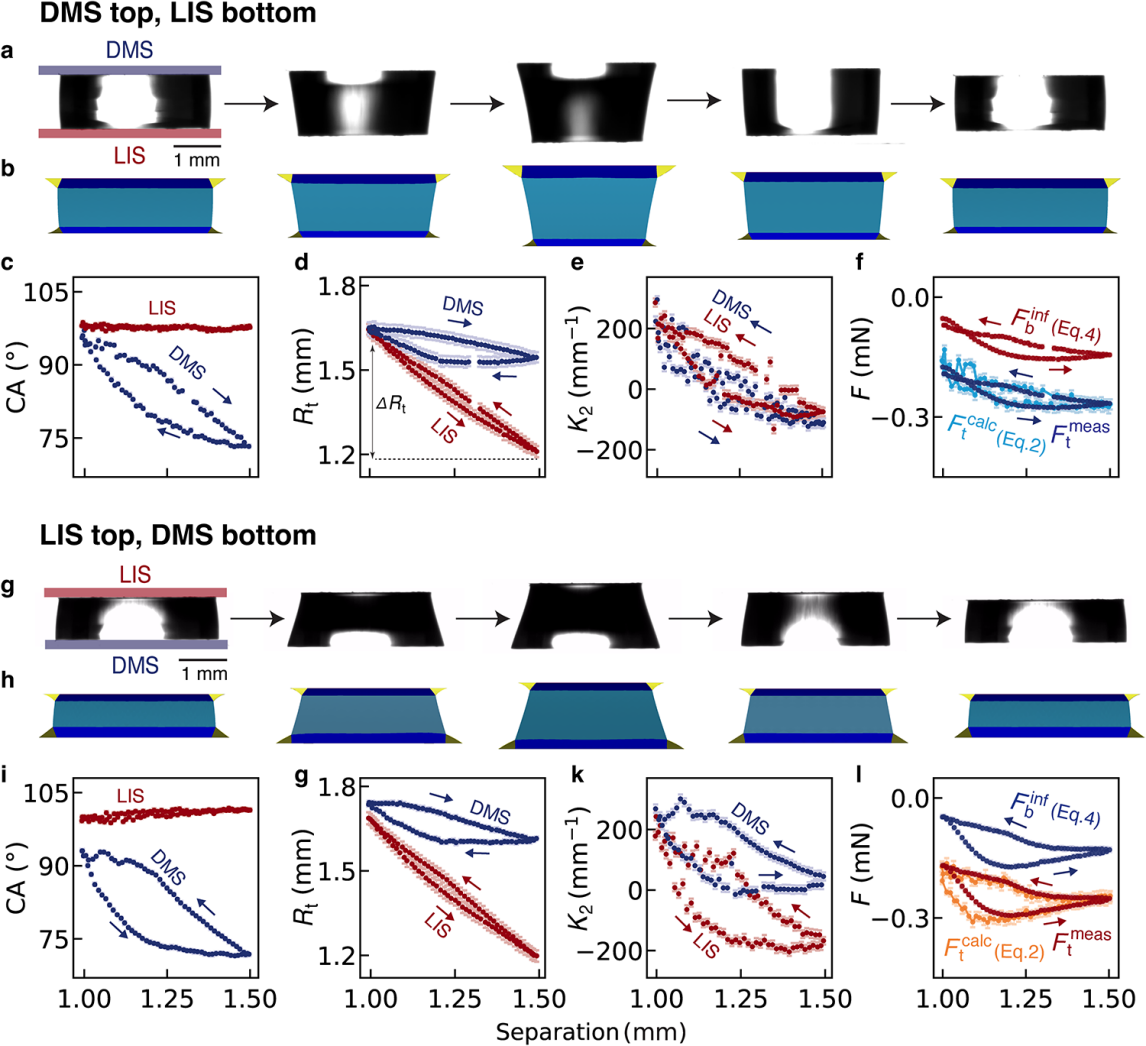
Comparative behavior of asymmetric capillary bridge between LIS (red traces) and DMS (blue traces). (a, g) Experimental images of bridge configurations during extension-compression. (b, h) Computer simulations incorporating the lubricant ridge (see the Methods section) are performed for both LIS and DMS, accounting for lubricant transport. The contact angles (c, i) remain almost constant on LIS, whereas DMS exhibits a pronounced hysteretic behavior, exploring a larger range of angles than that observed in the symmetric DMS system in Figure 3e. The evolution of capillary bridge–surface top contact radius *R*_t_ (d, g), meridional curvature *K*_2_ (e, k), and the exerted forces (f, l) are shown. Change in contact radius, denoted by Δ*R*_t_, is marked as an example in (d). Error bars represent standard errors.

The lubricant ridge is too small for its details to be directly resolved with our experimental setup, but its role can be assessed through computational simulations. This is achieved by introducing an oil meniscus around the three-phase contact region at both top and bottom surfaces. For the lubricant ridge on DMS, the oil–gas contact angle θ_og_ is set at 35° (inferred from Figure S5), while for LIS it is set to 15° (representing high spreading; the resulting behavior is very similar when lower contact angle is employed). Other relevant interfacial tensions are taken from measurements and derivation (see Computational model section and eq 5). To maintain symmetry and to balance computational costs and accuracy, the ridge volumes used in the simulations are larger than those in the experiments. This approximation is valid as long as the ridge remains much smaller than the capillary bridge itself, since the local Neumann balance at the three-phase contact line is preserved. As shown in Figure 6a–b,g–h, the simulated bridge geometries closely match the experimental images, confirming the importance of including lubricant ridges at both surfaces. Furthermore, the force calculated from the simulations using eq 6 agrees well with experimental measurements (Supporting Information SI 14). Overall, the consistency in both geometry and force demonstrates that the model in Figure 2 reliably captures the physics of capillary bridges involving lubricant ridges. Beyond the present application, this framework can be extended to describe liquid–liquid, liquid–solid, and three-phase interactions on functional surfaces.

We now further examine the geometrical and force responses in these two asymmetric systems. The radius of the contact area changes by Δ*R*_t_ = 0.5 mm at the LIS interface with no hysteresis, and by Δ*R*_t_ < 0.1 mm at the DMS interface with some hysteresis (Figure 6d,g). As expected, the bridge preferentially slides across LIS compared to DMS. When LIS is the bottom surface (with DMS on top), the meridional curvature *K*_2_ remains comparable near the top and bottom of the bridge (Figure 6e). This is expected, since the CAs are similar at the onset of the extension (∼95° for DMS and ∼98° for LIS). However, when LIS is positioned at the top and DMS at the bottom, the *K*_2_ at the two surfaces no longer match. An offset and distinct ranges of curvature values are observed near the top and bottom surfaces (Figure 6k). This effect can be explained by the influence of gravity on the capillary bridge geometry (CA and *R*_t_), amplified by enhanced lubricant transfer from LIS when it is on top.

Comparison of the measured, calculated and inferred capillary force shows consistently good agreement (Figure 6f,l), with larger hysteresis observed for the LIS-top-DMS-bottom system. In such system, gravity promotes oil transfer from the LIS top to the DMS bottom, leading to the formation of a large oil ridge that enhances pinning or friction during extension or compression of the bridge. Overall, the variations in force are comparable to those in the symmetric LIS systems (Figure 3l,o) regardless of the configuration, which can be explained by a combination of two factors. First, when LIS is present in the system, the surface tension of the bridge drops from ∼ 67 mN/m^55^ to ∼ 50 mN/m due to cloaking. Second, the LIS offers a nonpinning surface, allowing contact line to move preferentially and minimize the energy required to extend the bridge. Consequently, contact angles and contact radii follow similar trends on each surface in symmetric systems, whereas the radii of curvature and exerted force reveal the effects of oil transfer and friction characteristic of asymmetric bridges.

## Conclusion

In this study, we systematically investigate capillary bridges on LIS and compare the observed behavior with two “standard” noninfused solid surfaces: hydrophilic Glass and hydrophobic DMS. The good agreement between experiments, modeling, and theory demonstrates that our model accurately captures the behavior of capillary bridges in the quasistatic limit, including for LIS through the use of an apparent contact angle. In agreement with previous studies, contact line pinning is prevalent on Glass, giving rise to a complicated stick–slip motion; DMS exhibits typical contact angle hysteresis on hydrophobic surfaces, with well-defined geometrical features during extension and compression.^23,24,27,56^ In contrast, LIS^33,48,57,58^ exhibits markedly distinct trends due to the absence of pinning. First, no hysteresis is observed during bridge extension or compression. Second, the variation in capillary force is substantially reduced thanks to the frictionless nature of the lubricant. Third, the small forces and absence of pinning allow gravity to break the bridge symmetry, an effect that is often masked by pinning on solid surfaces. On LIS, gravity can affect the apparent CA by altering the pressure balance within the capillary, in agreement with theoretical predictions.^35,53^ Finally, lubricant cloaking on LIS^46–48^ reduces the effective surface tension and allows lubricant transport between the surfaces. The effect is most pronounced in asymmetric capillary bridges formed by a LIS and DMS, where lubricant transfer produces a ridge on the DMS surface, modifying capillary interactions and introducing localized pinning.

Further work will focus on dynamic interactions between the lubricant and capillary bridges, particularly the evolution of oil ridges over time under mechanical deformation and varying pressures. Incorporating dynamic effects, such as lubricant viscosity and the velocity of capillary bridge extension-compression, would extend the predictive capability of the model to practical applications, including printing, coatings, cell culture, and microfluidics. Overall, this study establishes a fundamental framework that can help design functional liquid-like surfaces with tunable and controllable capillary interactions.

## Supplementary Material





## Data Availability

All the data and videos used to create the figures presented in this paper as well as example simulation scripts are freely available at the Durham Research Online Repository: https://collections.durham.ac.uk/files/r26m311p40q (doi:10.15128/r26m311p40q)

## Notes

Details on surface characterization, experimental setup and imaging procedures, droplet placement, capillary bridge focusing and geometry analysis, reproducibility of measurements, gravitational effects, oil ridge formation and induced pinning on DMS, contact line pinning, effective surface tension for cloaked systems, fitting of bridges with oil ridges, lubricant transfer, and comparisons between experiments and simulations (PDF)
Oil ridge formation and induced pinning on DMS (AVI)
