# Supplementary material for: Stretching and Compressing Capillary Bridges on Hydrophilic, Hydrophobic, and Liquid-Infused Surfaces
Source: Langmuir. 2026 Jan 13;42(3):2561–71. doi: 10.1021/acs.langmuir.5c05016 (PMC12856891; doi:10.1021/acs.langmuir.5c05016)
Supplement: Supplementary file 1 [file la5c05016_si_001.pdf]

Supporting Information

Stretching and Compressing Capillary Bridges on Hydrophilic, Hydrophobic, and Liquid-infused Surfaces

Sarah Jane Goodband[1,⊥], Ke Sun[1,⊥], Kislon Voïtchovsky[1*], Halim Kusumaatmaja[2*]

[1]Department of Physics, Durham University, Durham DH1 3LE, UK

[2]Institute for Multiscale Thermofluids, School of Engineering, The University of Edinburgh, Edinburgh, EH9 3FB, UK

,⊥ These authors contribute equally

Correspondence:

* Email: kislon.voitchovsky@durham.ac.uk

* Email: halim.kusumaatmaja@ed.ac.uk

Table of contents

1. Surface topography characterization using atomic force microscopy (AFM) (Table S1)
2. Experimental setup and imaging system (Fig. S1)
3. Droplet placement for capillary bridge formation (Fig. S2)
4. Focusing on the top and bottom of capillary bridges (Fig. S3)
5. Reproducibility of extension-compression cycles (Fig. S4)
6. Acquisition of the radii of curvature
7. Impact of gravity
8. Oil induced pinning on DMS (Fig. S5, Video S1)
9. Contact line displacement over an extension-compression cycle (Fig. S6)
10. Effective surface tension for a cloaked capillary bridge
11. Fitting capillry liquid bridges with oil ridges (Fig. S7)
12. Capillry bridge stick–slip motion with contact line pinning (Fig. S8)
13. Lubricant transfer from LIS to DMS in an asymmetric capillary bridge system (Fig. S9)
14. Simulation and experiment comparison for LIS-top-DMS-bottom capillary bridge (Fig. S10)
15. References

1. Surface topography characterization using atomic force microscopy (AFM)

Table S1. AFM topographic characterization of glass and DMS surfaces. Imaging is performed in amplitude modulation using a Cypher ES AFM (Oxford Instruments, USA) at 25 °C in pure water. The AFM tip/cantilever (SNL-10 tip A, Bruker AFM Probes, USA, nominal spring constant of 0.43 N/m) was calibrated using its thermal spectrum after the measurements. Representative AFM images and the measured values (average ± standard deviation) are shown, obtained from three repeated measurements across randomly selected areas of each sample. The roughness statistics are processed using Gwyddion[1].

Material	AFM Topographic image	Image size	Mean roughness (S_a) in nm	RMS roughness (S_q) in nm	Surface area /projected area
Glass	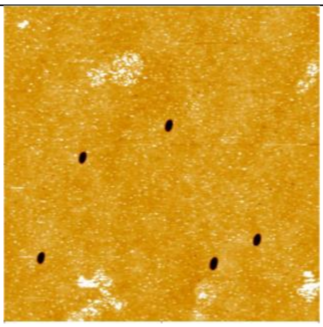	XY: 10 μm<br><br>Z: 10 nm (colour scale range)	0.449 ± 0.027	1.031 ± 0.207	1.00039 ± 0.00008
DMS	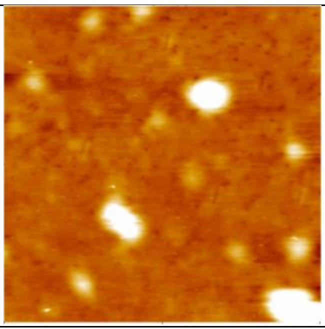	XY: 10 μm<br><br>Z: 30 nm (colour scale range)	2.297 ± 0.221	3.427 ± 0.380	1.00037 ± 0.00007

2. Experimental setup and imaging system

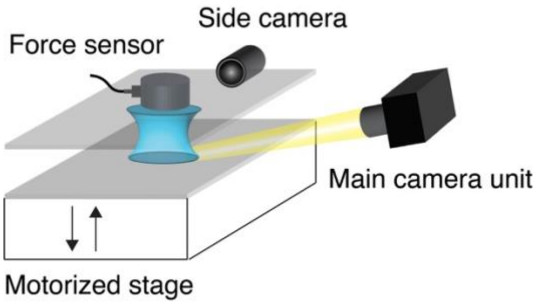

Figure S1. Schematics of the experimental setup and imaging system. A capillary bridge is formed between two parallel plates. The bottom plate is mounted on a motorized z-stage, which moves to compress or stretch the bridge at set speeds and distances, while the top plate is fixed and equipped with a force sensor to record the force on the upper surface. The system is imaged using a dual camera setup: a main high magnification camera unit (digital camera with zoom and magnifying lens) that focuses on the bridge edges to extract geometrical parameters (contact angles, contact radii, and bridge profile). Here, the focus is set on the bottom edge of the bridge as an example; during measurements, the top edge is similarly imaged when extracting the associated parameters (see SI 4 for details). A secondary side view camera provides a complementary full profile view of the bridge. The z-stage, force sensor, and both cameras operate in synchrony. Further details of the apparatus can be found in Goodband et al.[2]

3. Droplet placement for capillary bridge formation

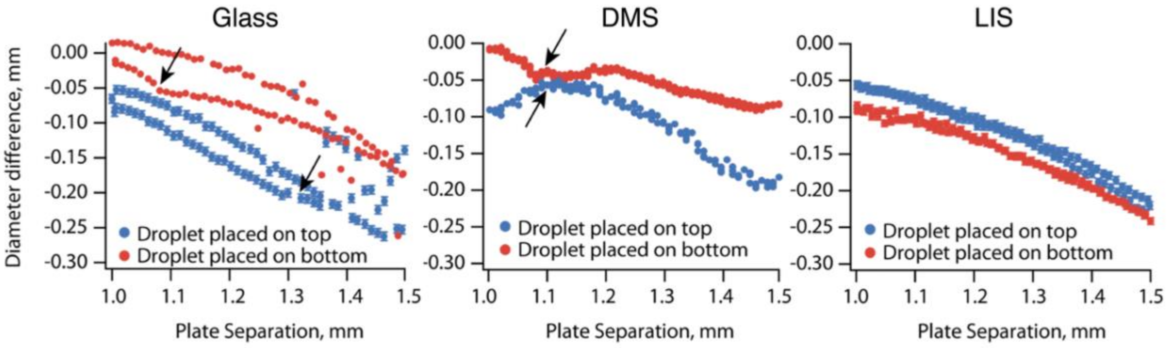

Figure S2. Variation of the difference between the top and bottom contact diameters of the capillary bridge over an extension-compression cycle. In all cases, a 10 µL droplet of glycerol solution is initially placed either on the top surface (blue) or bottom surface (red) before forming a capillary bridge with the other surface. The difference is negative for most surface separations, indicating a larger bottom diameter. This consistent with the expected effect of gravity. However, on Glass, the difference can initially be positive at small separation, depending on where the droplet is initially placed. Additionally, for Glass and DMS, the largest different is observed when the droplet is initially placed on the top surface. These observations are counterintuitive considering how gravity breaks the capillary bridge symmetry and point to pinning effects. On both Glass and DMS, significant pinning events can be seen during the experiment (see arrows). On LIS, the system behaves consistently as expected when considering gravity and in the absence of pinning. The droplet position at start does not affect the final capillary bridge behavior.

4. Focusing on the top and bottom of capillary bridges

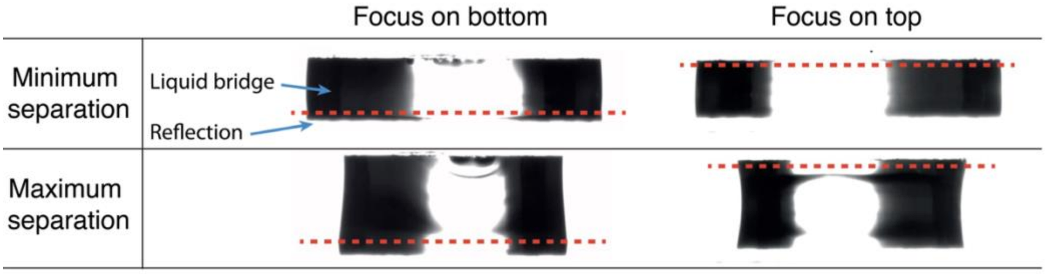

Figure S3. Example of sequential measurements being taken at the bottom (left) and top (right) surfaces. In order get a better view of the capillary bridge–surface contact region, the camera is placed at an angle.[2] This makes it is challenging to simultaneously and accurately measure the top and bottom of the bridge (assuming a 1 camera setup). For experiments that require information from both extremities, the measurements are conducted sequentially as illustrated here: multiple extension-compression cycles are obtained focusing on the bottom of the bridge (left) and subsequently on the top interface (right) of the same capillary bridge (see the next Supporting Information SI 5 for confirmation of the data reproducibility). The above example is taken on DMS and the bridge can be seen to have a reflection on either the top or bottom surface. A red line denotes the contact line of the bridge with the surface. This allows for an accurate detection of the bridge intersection with the surface, provided it is in focus.

5. Reproducibility of extension-compression cycles

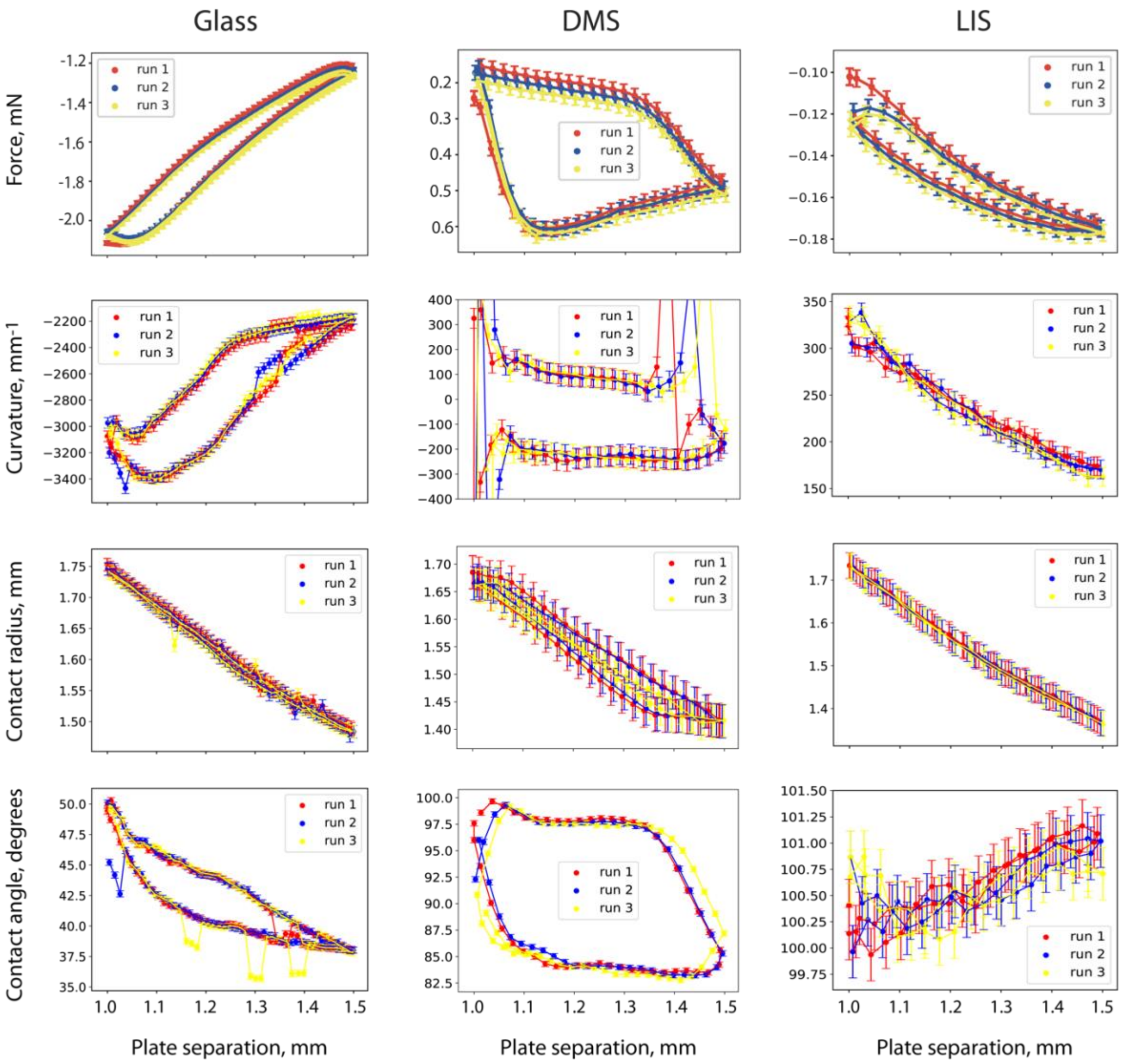

Figure S4. Evolution of the force, curvature, contact radius and contact angle for each system over a number of extension-compression cycles. In all cases the camera is focusing on the top part of the bridge. For each set of surfaces, the measurements are fully reproducible within error between consecutive cycles. This confirms the suitability of a sequential strategy for investigating the behaviour of the bottom and the top of a particular capillary bridge.

6. Acquisition of the radii of curvature

The radii of curvature are obtained at the plate (top or bottom) where the capillary force is being calculated. Two components are measured: the meridional radius of curvature, R_2, and the azimuthal radius of curvature, R_1. The detailed method for processing the experimental data can be found in Goodband et al.[2],and a similar calculation was employed by Wang et al.[3] Here, we briefly summarize the procedure as follows. To obtain the meridional radius of curvature (R_2), the local edge of the capillary bridge is fitted to a second-order polynomial. From this fit, the meridional curvature is computed as:[4]

$$K_2 = \frac{\left|\frac{d^2y}{dx^2}\right|}{(1 + (\frac{dy}{dx})^2)^{3/2}}$$

and the corresponding meridional radius of curvature is simply $R_2 = \frac{1}{K_2}$.

The azimuthal radius of curvature (R_1) is obtained from geometric relations at the three-phase contact line. At the top and bottom plates, respectively:

$$\frac{1}{R_1} = \frac{\sin\theta_t}{R_t} \quad \text{and} \quad \frac{1}{R_1} = \frac{\sin\theta_b}{R_b}.$$

Here, R_t and R_b are the top and bottom contact radii, and θ_t and θ_b are the corresponding contact angles as shown in Fig. 1 of the main text.

7. Impact of gravity

The capillary bridge used in this study are several millimeters wide for a height varying between 1 and 1.4 mm. This is relatively close to the capillary length of the bridge's solution (2.39 mm). Alternatively, we find the bond number B_o of the capillary bridge at $0.1 < B_o < 0.4$, relatively close to 1. Gravitational effects must therefore be taken into account not only in terms of additional weight on the bottom surface, but also for its deformation of the bridge geometry.

Bond number calculation

The Bond number (B_o) is defined as the ratio of gravitational to surface tension forces with a Bo value of less than one indicating that surface tension dominates over gravity. It is given by the following equation:

$$B_o = \frac{gL^2(\rho_L - \rho_g)}{\sigma}$$

where g is the acceleration due to gravity (9.81 ms^{-2}), L is the characteristic length of the system (here taken as the bridge height[5]), ρ_L is the liquid density, ρ_g is the gas density, and σ is the surface tension of the liquid. For the present study we find B_o = 0.175 for a capillary bridge at minimum extension (1 mm), and B_o = 0.394 for a capillary bridge at maximum extension (1.5 mm). While both B_o numbers are smaller than 1, they indicate that gravitational effects still play a role that should be carefully considered in this setup.

8. Oil induced pinning on DMS

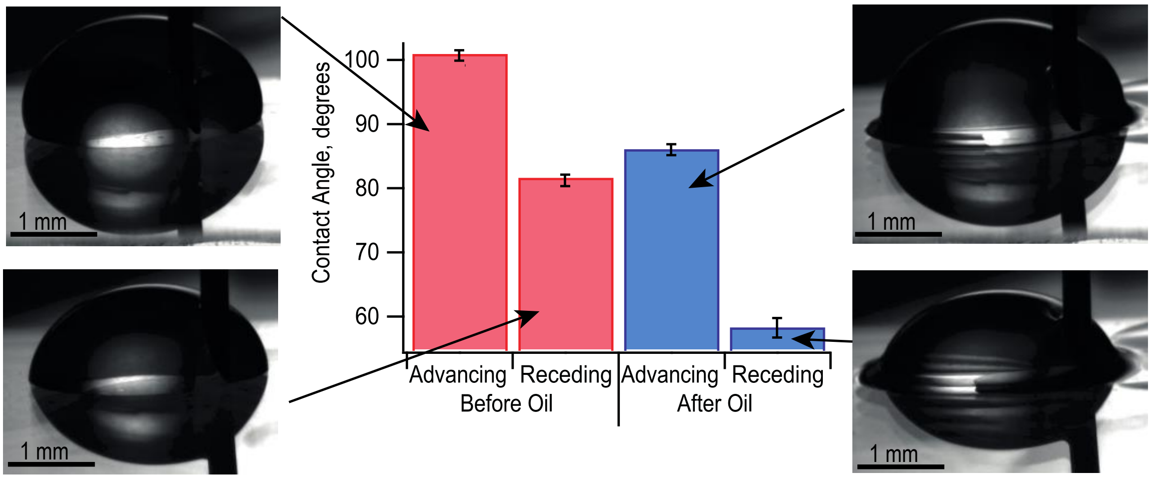

Figure S5. Silicone oil-induced droplet pinning on DMS. Representative advancing (top) and receding (bottom) contact angles are shown before (left) and after (right) the lubricant (silicone oil) contacted the base of a droplet of glycerol solution. As the oil contacts the base of the droplet, it spontaneously cloaks it (see Supplementary video S1) resulting in a reduction of both the advancing and receding angles. The decrease is however more marked for the receding angle (histograms) due to pinning of the contact line by the oil ridge. The ridge forces the droplet to reduce its CA with the DMS as liquid is being pumped out of the droplet. The whole experiment can be visualised as a video (Supplementary video S1). The analysis software developed for extracting geometrical parameters from the capillary bridges[2] cannot be applied here and the CA measurements were done manually using the angle tool in ImageJ[6]. Comparative testing on sample capillary bridge images typically evidenced a 2 degrees discrepancy between these two methods, with the capillary bridge fitting method in being more accurate on capillary bridges[2]. This effect, together with the experimental variability (see Methods section) explain the difference in CA between this figure and Figs. 5 and 6 of the main text. Each set of data is however consistent within itself, with the error bars shown representing two standard errors.

Video S1: Behaviour evolution of a droplet of glycerol solution placed on a DMS surface as silicone oil (the lubricant) diffuses towards the droplet base. In order to illustrate the advancing and receding CAs of the droplet with the DMS, 50 µL of the glycerol solution is constantly pumped in and out of the droplet using a motorised syringe pump. The pumping in (out) of the glycerol solution follows a linear ramp. A 40 µL drop of silicon oil is deposited on the DMS surface, several millimetres away from the drop of glycerol solution and allowed to diffuse in all directions. As the oil contacts the drop, it immediately and spontaneously cloaks it resulting in a change of the apparent advancing and receding CAs.

9. Contact line displacement over an extension-compression cycle

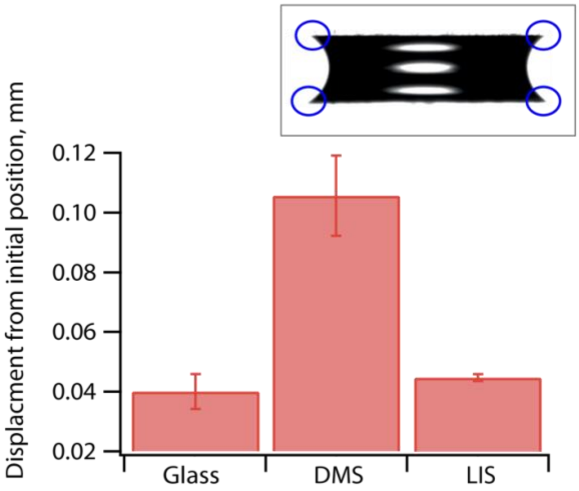

Figure S6. Variation of contact line position between the beginning and the end of a complete extension-compression cycle. The data represents the average displacement of the contact line over 4 separate points (blue highlights in the inset image). On Glass, very little displacement occurs due to high pinning (as shown in Fig. 3g of the main text and Fig. S4 of SI 5), the contact angle constantly changes. The measurement uncertainty represents the uneven movement of the contact lines, where one side may preferentially move more than the other. On DMS, the capillary bridge experiences pinning and a large displacement (sliding). Uneven movement caused by pinning leads to greater displacement and larger errors. In contrast, on LIS, the capillary bridge slides very slowly during the course of the measurement, with all contact lines moving at the same rate, hence resulting in a small error.

10. Effective surface tension for a cloaked capillary bridge

Deriving an effective surface tension for a droplet or liquid bridge which is cloaked is not straightforward because the effective surface tension of a cloaked liquid is known to change with the thickness of the cloaking thin film[7]. In our system, the lubricant film on the bridge cannot be measured directly. Furthermore, the film thickness may be non-uniform and can evolve dynamically, including through lubricant transport between the two surfaces via the capillary bridge. To estimate an effective surface tension, we therefore employ the cloaking film approximation:[8]

$$\gamma_c = \gamma_{dl} + \gamma_{la}$$

where γ_c is the effective (cloaked) surface tension, γ_{dl} is the droplet–lubricant interfacial tension, and γ_{la} is the lubricant–air surface tension. For our system, $\gamma_{dl} = 27.6\ mN/m$ for an 80 wt% glycerol–water droplet in contact with 20 cSt silicone oil, and $\gamma_{la} = 20.6\ mN/m$ for 20 cst silicone oil in air, both measured using the pendant drop method[9,10]. This yiedls $\gamma_c = 48.2\ mN/m$. An approximate sum from literature value[8] for a similar system gives $\gamma_c = 52.9\ mN/m$. Averaging these two estimates, we adopt an effective droplet (cloaked)–gas surface tension of

$$\gamma_{dg} \sim 50\ mN/m.$$

11. Fitting capillary liquid bridges with oil ridges

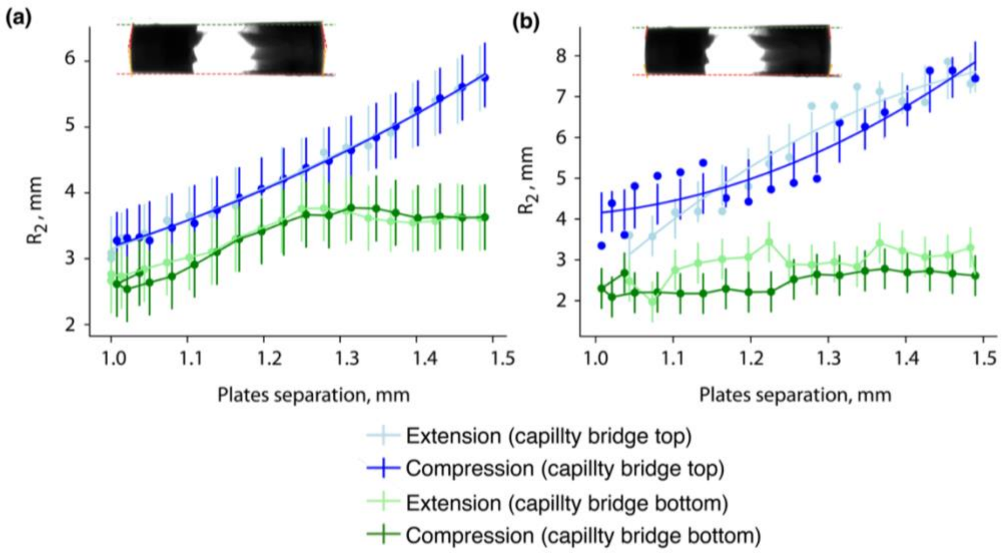

Figure S7. Impact of the selected region of the capillry bridge profile used in the fitting[2] carried out to derive the bridge's meridional radius of curvature R_2. To illustrate the issue, the upper graph (a) shows the curvature derived from fitting half of the bridge's height, whereas only a quarter of the bridge's height is taken in the lower graph (b). In each graph, the curvature is shown for both the top surface (blue) and bottom surface (green) with the extension data shown darker than the retraction in order to aid visualisation. The example is taken for a capillry bridge between two LIS surfaces. The error bars on all curves represent 0.5 mm.

12. Capillary bridge stick–slip motion with contact line pinning

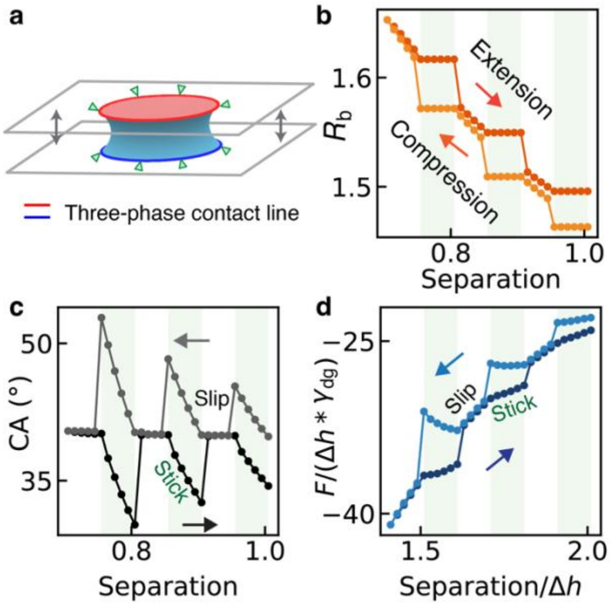

Figure S8. Simulation of the stick–slip contact line motion on a non-ideal surface. Here, this is achieved by employing three-phase contact line pinning and release during a capillary bridge compression-extension cycle, as illustrated in (a). The green shaded area indicates where the contact line is pinned, and white area indicates where the contact line is free to move. The change in the bridge bottom radius R_b is shown in (b), the measured contact angle close to the bottom contact line is shown in (c), and the normalized measured capillary force through simulation is shown in (d).

To investigate the generality of the stick–slip behavior, we explored an alternative scenario of contact line pinning and depinning. We introduced pinning and release of the three-phase contact line on both plates simultaneously in an extension-compression cycle (Fig. S8a). When the contact line is free to move, the bottom contact radius R_b increases during compression and decreases during extension (Fig. S8b), with the measured CA near the contact line remaining constant and close to the input parameters (Fig. S8c). When the contact line is pinned, the bottom radius remains unchanged (Fig. S8b), and the CA increases during compression and decreases during extension as the plates move (Fig. S8c). The change in CA is more pronounced at smaller plate separation, where the bridge is more compressed and constrained such that pinning triggers a stronger response. The capillary force in the simulation was calculated by Eq. 1 with the pressure obtained from the simulation model and normalized by the product of the plate separation change Δh and the droplet–gas interfacial tension γ_{dg}. The resulting force (Fig. S8d) exhibits a stepwise behavior similar to that visible for Glass in Fig. 3j and that simulated in Fig. 4e for chemically heterogeneous surfaces.

13. Lubricant transfer from LIS to DMS in an asymmetric capillary bridge system

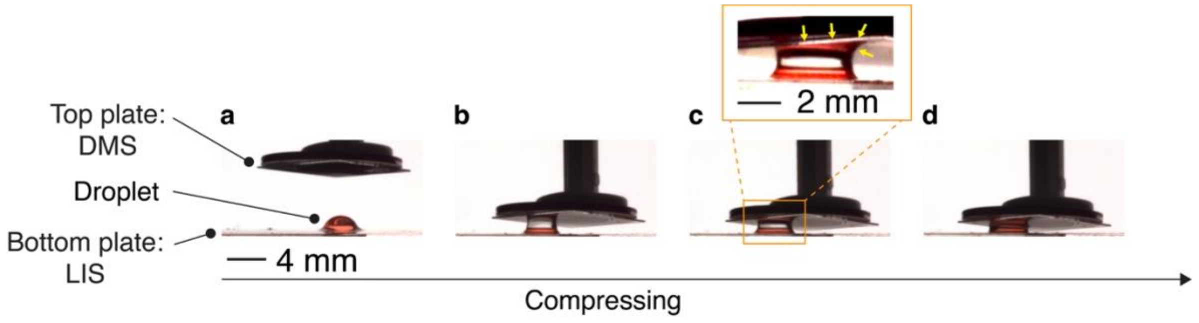

Figure S9. Formation and compression of a capillary bridge between a dyed silicone-oil-infused LIS (bottom plate) and a DMS surface (top plate). The lubricant infused into the LIS is 20 cSt silicone oil dyed with Oil Red O; this dyed lubricant visibly cloaks the deposited droplet (10 µL, 80 wt% glycerol in water), as shown in (a). The bottom plate is then raised to form a capillary bridge, with a dyed oil ridge present at the LIS–bridge contact. Upon compression (b–d), an oil ridge forms at the bridge contact with the top DMS surface, highlighted by yellow arrows in the inset of (c). Note that an oil ridge is also present in (b), though it may not be clearly visible due to the viewing angle. At smaller plate separations (d), the entire bridge becomes cloaked by the dyed lubricant, indicating that the lubricant has reached and interacted with the top DMS surface.

To directly examine whether lubricant transfers from the LIS to the DMS surface in the asymmetric capillary bridge configuration, we conduct a visualisation experiment using a dyed silicone oil infused into the LIS. The goal is to qualitatively assess lubricant migration independent of gravity-driven effects. The dyed lubricant is prepared by dissolving 1 wt% Oil Red O in 20 cSt silicone oil, assisted by sonication and filtered through a 0.2 µm syringe filter to remove undissolved dye. This lubricant is then infused into the LIS. Owing to the limited sensitivity of the digital camera, the LIS is loaded with ~50% more dyed lubricant than the standard protocol to ensure that the oil ridge was optically detectable.

A droplet is deposited onto the dyed LIS, where it becomes visibly cloaked by the lubricant. The bottom (LIS) plate is then brought into gentle contact with the top DMS substrate to form a capillary bridge, during which a dyed oil ridge is clearly visible on the LIS side. When the bridge was compressed to heights comparable to those studied in the main text (~1.72 mm in Fig. S9c and ~0.94 mm in Fig. S9d; the relevant range in the main text is ~1–1.5 mm), an oil ridge also form at the bridge–DMS contact (Fig. S9c, highlighted with yellow arrows in the inset). At smaller separations, the entire bridge gradually turned red (Fig. S9d), indicating that the dyed lubricant cloaked the full bridge interface.

This experiment is qualitative: the film thickness and detailed lubricant flow could not be resolved with the current optical setup, and the manual operation involved higher velocities ($\gg$ 0.008 mm s^{-1} used elsewhere in this work), and increased lubricant loading compared to the main experiments. Nevertheless, these observations support the interpretation that lubricant exchange occurs during asymmetric capillary bridge deformation. Quantitative characterisation will require higher resolution techniques such as confocal microscopy with fluorescent dyes, something beyond the scope of the current work.

14. Simulation and experiment comparison for LIS-top-DMS-bottom capillary bridge

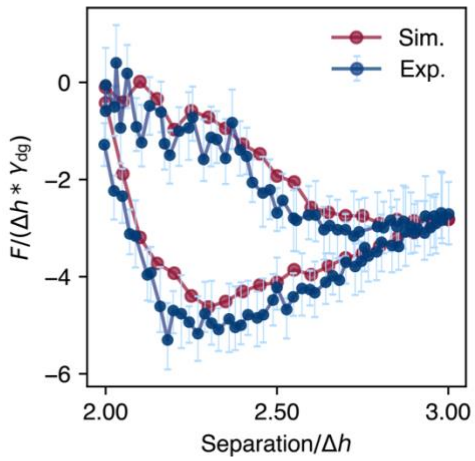

Figure S10. Normalized force versus separation for a capillary bridge with the LIS at top and DMS at bottom. The simulated force is obtained from the capillary bridge pressure output following Eq. 1 of the main text, while the experimental force is inferred at the bottom surface using Eq. 4 of the main text. The good agreement between experiments and simulations indicates that the model accurately captures the behavior of asymmetric capillary bridges.

15. References

(1) Nečas, D.; Klapetek, P. Gwyddion: An Open-Source Software for SPM Data Analysis. *Cent. Eur. J. Phys.* **2012**, *10* (1), 181–188.

(2) Goodband, S.; Kusumaatmaja, H.; Voïtchovsky, K. Development of a Setup to Characterize Capillary Liquid Bridges between Liquid Infused Surfaces. *AIP Adv.* **2022**, *12*, 015120.

(3) Wang, Q.; Chen, W.; Wu, J. Effect of Capillary Bridges on the Interfacial Adhesion of Wearable Electronics to Epidermis. *Int. J. Solids Struct.* **2019**, *174–175*, 85–97.

(4) do Carmo, M. P. *Differential Geometry of Curves and Surfaces: Revised and Updated Second Edition*; Courier Dover Publications, 2016.

(5) Radoev, B.; Petkov, P.; Ivanov, I. Capillary Bridges — A Tool for Three-Phase Contact Investigation; IntechOpen, 2015; p 32.

(6) Schneider, C. A.; Rasband, W. S.; Eliceiri, K. W. NIH Image to ImageJ: 25 Years of Image Analysis. *Nat. Methods* **2012**, *9* (7), 671–675.

(7) Pepper, K. G.; Bahrim, C.; Tadmor, R. Interfacial Tension and Spreading Coefficient of Thin Films: Review and Future Directions. *J. Adhes. Sci. Technol.* **2011**, *25* (12), 1379–1391.

(8) Gunjan, M. R.; Kumar, A.; Raj, R. Cloaked Droplets on Lubricant-Infused Surfaces: Union of Constant Mean Curvature Interfaces Dictated by Thin-Film Tension. *Langmuir* **2021**, *37* (22), 6601–6612.

(9) Berry, J. D.; Neeson, M. J.; Dagastine, R. R.; Chan, D. Y. C.; Tabor, R. F. Measurement of Surface and Interfacial Tension Using Pendant Drop Tensiometry. *J. Colloid Interface Sci.* **2015**, *454*, 226–237.

(10) Daerr, A.; Mogne, A. Pendent_Drop: An ImageJ Plugin to Measure the Surface Tension from an Image of a Pendent Drop. *J. Open Res. Softw.* **2016**, *4* (1), e3.
